# Supplementary material for: Prenatal Caffeine Exposure Is Linked to Elevated Sugar Intake and BMI, Altered Reward Sensitivity, and Aberrant Insular Thickness in Adolescents: An ABCD Investigation
Source: Nutrients. 2022 Nov 3;14(21):4643. doi: 10.3390/nu14214643 (PMC9658384; doi:10.3390/nu14214643)
Supplement: Supplementary file 1 [file nutrients-14-04643-s001.zip › nutrients-1918030-supplementary.pdf]

Table S1. Associations Between Prenatal Caffeine Exposure, and Gram Intake of Sugar Sources.

Food Items (gm)	Daily vs. No (β; 95% CI; *p*)	Weekly vs. No (β; 95% CI; *p*)	Less than Weekly vs. No (β; 95% CI; *p*)
soft drinks	30.37; 21.45 – 39.29; <0.001*	12.77; 3.47 – 22.07; 0.007	8.92; -1.08 – 18.93; 0.08
Applesauce Canned fruit	-2.41; -4.19 – -0.64; 0.008	-1.59; -3.45 – 0.25; 0.09	-1.69; -3.69 – 0.30; 0.09
Fruit juice	3.56; -4.25 – 11.38; 0.37	-4.96; -13.12 – 3.19; 0.23	-0.41; -9.19 – 8.37; 0.92
Apple banana orange (Fruit)	-2.34; -6.61 – 1.91; 0.28	-1.43; -5.88 – 3.01; 0.52	-3.85; -8.64 – 0.93; 0.11
Any other fruit	-1.81; -4.84 – 1.20; 0.23	-1.83; -4.99 – 1.31; 0.25	0.39; -2.99 – 3.79; 0.81
Ketchup salsa	0.14; -0.11 – 0.39; 0.26	0.04; -0.21 – 0.31; 0.73	-0.005; -0.29 – 0.28; 0.97
Ice-cream	1.08; -1.29 – 3.45; 0.37	1.27; -1.20 – 3.75; 0.31	1.96; -0.70 – 4.63; 0.15
Candy bars	0.28; -0.17 – 0.74; 0.23	-0.31; -0.79 – 0.16; 0.19	0.24; -0.26 – 0.76; 0.34
Cookie donut cake	0.75; 0.03 – 1.48; 0.03	0.52; -0.23 – 1.27; 0.17	0.66; -0.14 – 1.47; 0.11
Cereal with sweet	0.48; -0.25 – 1.22; 0.19	0.32; -0.44 – 1.09; 0.41	-0.22; -1.05 – 0.60; 0.59
Milk chocolate	4.35; 0.19 – 8.5; 0.04	0.19; -4.1 – 4.53; 0.93	2.3; -2.31 – 7.03; 0.32
Breakfast protein bars	-0.10; -0.95 – 0.74; 0.80	-0.01; -0.90 – 0.87; 0.97	-0.37; -1.33 – 0.57; 0.43
Wholewheat bread	-0.83; -1.75 – 0.07; 0.07	-0.27; -1.23 – 0.68; 0.57	-1.20; -2.23 – -0.17; 0.02

Note: Here, *denotes significance that survived Bonferroni-correction for multiple comparisons with $p < 0.05$.
